# Nestlings reduce their predation risk by attending to predator-information encoded within conspecific alarm calls

**DOI:** 10.1038/s41598-017-11528-y

**Published:** 2017-09-15

**Authors:** Ahmad Barati, Paul G. McDonald

**Affiliations:** 1 Avian Behavioural Ecology Laboratory, Zoology, University of New England, Armidale, NSW 2351 Australia; 2 Department of Environment, Malayer University, Malayer, Iran

## Abstract

Predation is one of the main threats to altricial nestlings, with predators often locating nests via eavesdropping on begging signals. Nestlings may be able to adjust their begging based on the current level of risk by monitoring both intra- and interspecific alarm calls near the nest. We show that noisy miner (*Manorina melanocephala*) nestlings can differentiate between terrestrial and aerial alarm calls of their own species, as they suppressed begging behaviour for longer in response to terrestrial rather than aerial alarm calls. This differential response is potentially due to greater danger that terrestrial calls encode. In contrast, nestlings ignored alarm calls of the sympatric grey butcherbird (*Cracticus torquatus*) and continued to beg but reduced begging intensity in response to the non-alarm calls of a sympatric eastern rosella (*Platycercus eximius*), suggesting nestlings were likely responding based upon similarity to a known signal as opposed to expressing a learnt behaviour. Results show that nestlings respond adaptively to two different intraspecific alarm signals but have not learnt to respond to the alarm calls of sympatric species. These suggest that nestlings are able to take advantage of the complex vocal repertoire that adults produce, although discernment is an issue when filtering out irrelevant stimuli.

## Introduction

In many animal taxa, offspring produce begging signals to solicit food from parents^1^. In birds, solicitation displays usually incorporate begging calls, particularly in altricial nestlings where offspring are totally dependent on parents for their nutrition and needs^2^. These begging calls act as proximate cues of need, with parents and even nest attendants in cooperative systems adjusting their feeding effort to begging intensity and its acoustic components^2,3^. Although increased begging calls can provoke higher provisioning rates by parents, begging calls are also costly to nestlings. Two main costs of begging for nestlings are an increased metabolic rate^4^ and an increased risk of predation^2^. Whilst metabolic costs associated with the escalation of begging seem insufficient to prevent their increase^5^, enhanced predation risk with greater levels of signal appears to be the main cost of begging through increased attraction of predators to the nest^3,6^.

One way by which the predation risk associated with begging signals could be mitigated by nestlings is through responding appropriately to parental alarm signals^7–10^. In many taxa, parents use alarm calls to warn offspring of the presence of danger^11–13^. This parent-offspring communication is particularly important for altricial species in which nestling are incapable for moving and are highly dependent on care provided by parents and cannot physically escape predators and typically lack the ability to recognise them for much of their development^14^.

In addition to warning nestlings generally, parental alarm calls can encode information on the type and urgency of predators^15,16^, which can allow nestlings to respond in the most adaptive manner^16^. ‘Mobbing’ calls are given by many species to defend nests against stationary predators posing an immediate threat to young, while in some species a different alarm call is given to signal potential danger such as a flying raptor^17–20^. The way young animals respond to these different alarm calls is thought to be adaptive and is associated with differences in the type and level of predation risk^16,21^. For example, in California ground squirrels (*Spermophilus beecheyi*), juveniles show stronger responses to alarm calls for ground predators than they do to those targeting aerial predators, most likely as a consequence of differential predation risk^21^. In japanese great tits **(** *Parus major* **)**, different parental alarm signals elicit specific responses to predators in young. Nestlings either leave the nest hollow in response to alarm calls that are given for snakes, or crouch down into the nest in response to alarm calls given to corvids^13^.

Further, nestlings typically live in environments where they could potentially eavesdrop on the alarm calls of other species to further mitigate their predation risk. This eavesdropping on the alarm cues or signals of other species may well provide an important opportunity to acquire additional predator information, either through innate mechanisms or learnt responses^22^. Innate responses to interspecific alarm calls are most likely if calls are similar between taxa^23^, while conversely learnt responses most easily arise through personal experience or social learning from other nestlings^24^. In these previous studies, research was conducted at a time when the offspring were able to respond with movement. However, for altricial birds, movement away from the nest is not possible for much of their development. Given this, an appropriate adjustment of begging following alarm calls of the same species may be particularly important to enhance nestling survival^25^. We know that parental alarm calls might suppress nestling vocalisations^8^, however, how nestlings adjust their vocalisations in response to different intraspecific alarm call signals is not currently well known.

**Figure 1:**
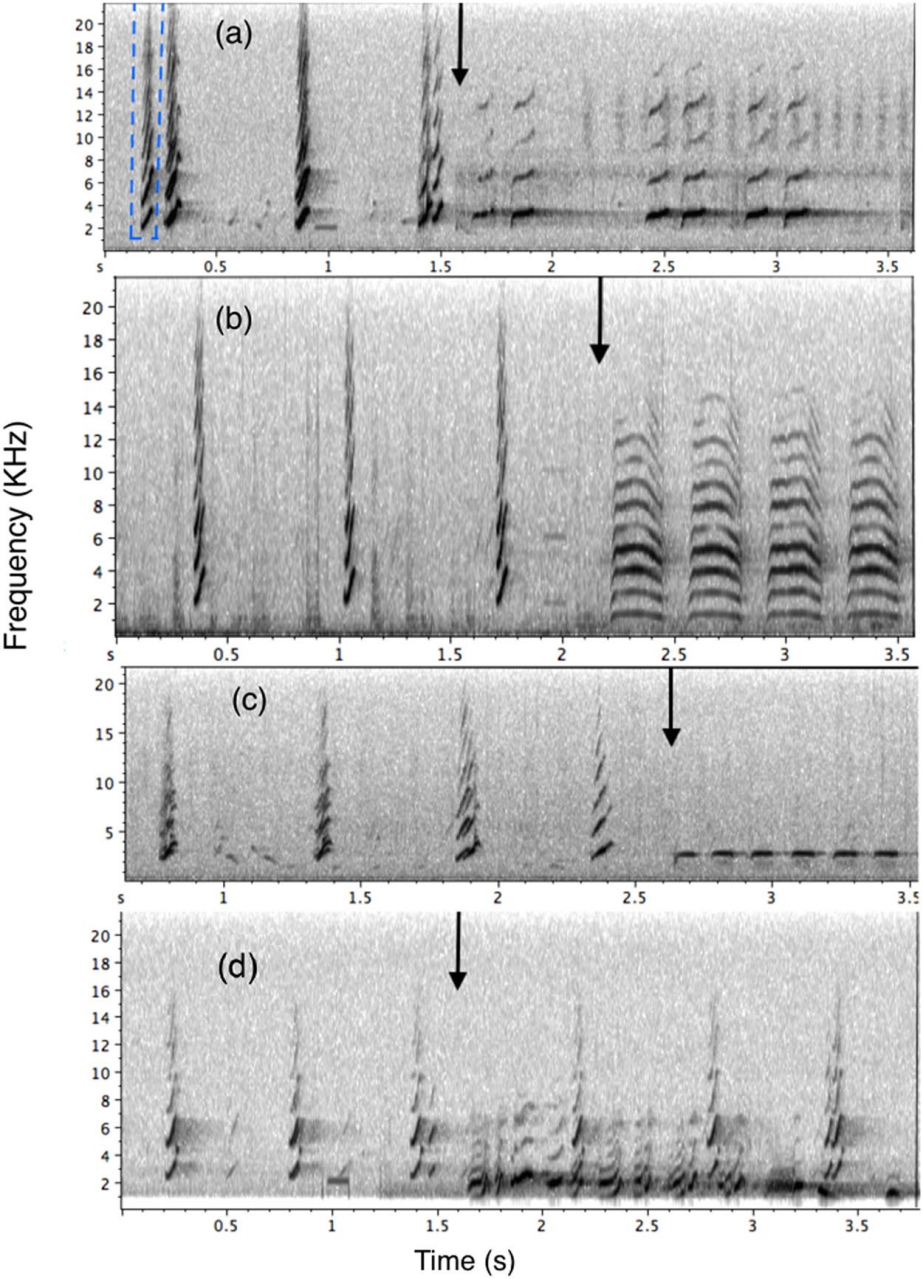
Spectrograms of exemplars of nestling noisy miners responding to different playback stimuli. Nestlings suppressed vocalisations when they heard intraspecific aerial (**a**) or chur alarm calls (**b**) and sympatric rosella chatter calls (**c**) but kept begging after playback of the sympatric butcherbird alarm call (**d**). On each spectrogram the time that playback commenced is indicated with arrows. An example begging syllable produced by the nestlings is highlighted by the dashed line (**a**).

The aim of this current study was to understand the mechanism by which nestling noisy miners (*Manoriana melanocephala*), an altricial species, respond to functionally referential (i.e. acoustically different calls that elicit different anti-predator behaviour responses in adult birds) intraspecific alarm calls in comparison to familiar interspecific alarm calls. Noisy miners are an Australian honeyeater with open cup nest that is often predated by a range of predators^26,27^. Noisy miners further possess a complex acoustic repertoire^28^ and produce two functionally referential alarm calls for different predatory types^20^: (1) Aerial alarm calls, which include a series of high-pitched, up-slurred whistles (Fig. 1a), are given in response to aerial predators, primarily brown goshawks (*Accipiter fasciatus*), and pied currawongs (*Strepera graculina*), in the study area^20,28^. (2) Terrestrial alarm calls (hereafter chur call), a vocalisation with a broad frequency and multiple harmonics (Fig. 1b) that is given to ground-based or perched threats and attracts other miners to the area^28–30^. These two types of alarm call are functionally referential and elicit different anti-predator behaviour responses in adult birds tested in both the field and under controlled laboratory conditions. For example miners give aerial alarm calls when they detect a flying raptor and then change to the chur call when the same predator is perched^20^. Both types of predators (e.g. terrestrial and aerial) pose a threat to nestlings, and may potentially use the conspicuous vocalisations nestlings produce to locate them^3^. Ceasing vocalisation production when these predators are near would therefore be beneficial for nestlings and may help mitigate their predation risk.

In addition to intraspecific alarm calls, nestling noisy miners are frequently exposed to two different interspecific calls at the focal study sites. First, the grey butcherbird (*Cracticus torquatus*), is common in noisy miner colonies and produces a distinct alarm call in response to potential threats to the nest^26^. Given that butcherbirds are closely associated with noisy miners and also give alarm calls when exposed to similar predators as miners^26,31^, nestling noisy miners would also benefit from ceasing vocalisation production in response to butcherbirds alarm calls. Second, the chatter calls of the eastern rosella (*Platycercus eximius*) are also commonly heard in the study areas, providing a non-alarm control vocalisation. This system therefore provided an opportunity to compare nestling responses to two intraspecific and two interspecific calls. We predicted that: (1) nestlings would supress begging and vocalisations in response to hearing intraspecific alarm calls in order to reduce their risk of being overheard by potential predators; (2) nestlings would respond more strongly to terrestrial intraspecific alarm calls as they encode greater danger than aerial alarm calls; (3) nestlings may have learnt to respond to the pertinent alarm calls of the butcherbird, but not the irrelevant rosella vocalisations.

## Methods

### Study population and general methods

The focal populations of this study were two colonies of noisy miners located at Newholme Field Research Station of the University of New England (30° 25′ 24″S; 151° 38′ 84 38″E) and Dumaresq Dam (30°30′S, 151°40″E), located 10 and 12 km, respectively, north-west of Armidale, NSW, Australia. The most common canopy species in these areas is New England peppermint (*Eucalyptus nova-anglica*)^32^, with sparse understorey vegetation dominated by introduced pasture grasses, consistent with the typical habitat of noisy miners^26^. This study was carried out in accordance with the approved (Protocol AEC13–142) guidelines and regulations of University of New England, Animal Ethics Committee. Fieldwork was conducted in the 2015 breeding season from mid-September to late October. To find nests, we surveyed study sites every 2–3 days for signs of nesting activity from mid-August when nesting activities normally commence at these colonies^27^. Once nest sites were located, they were marked with small numbered cattle ear tags (Allflex, Australia) attached to a nearby tree. We checked the contents with direct observation (using a ladder) or by a mirror attached to the end of a 10-metre pole to determine hatching dates. We noted the hatching date for each nest, and monitored the brood at least every other day. To assess nestling status (e.g. survived, predated) whilst minimising disturbance, we used the same methods as outlined above, but once nestlings’ vocalisations were audible from the ground (approximately 5 days post-hatch), we used this as a cue to their status. Broods were monitored until experiments were conducted at the age of 14 days, close to fledging age, which is usually15 days post hatch in our study populations.

### Stimuli recording and preparation

Noisy miners’ chur and aerial alarm calls were recorded between September 2013 and October 2015. Chur calls were given to approaching humans (N = 17 individuals), while adult miners were provoked to give aerial alarm calls by throwing a hat in the air nearby (N = 10 individuals). Vocalisations emitted by individuals were recorded at 44.1 kHz using a portable Professional Solid State Recorder (Marantz PMD661, Japan) with 16-bit accuracy in uncompressed wav format using a Sennheiser shotgun microphone (ME67, USA), protected by a fur windshield (Rycote Softie, UK). Recordings were conducted in standard condition in terms of distance to caller (~15 m) and weather conditions (i.e. no wind) to ensure a high signal to noise ratio in recordings. The identity of the focal bird was determined using the unique leg colour bands fitted to each adult in these colonies using binoculars (10 × 42, Nikon, Japan) or a telescope (Gerber Montana 15–45x). The amplitude of five exemplars of each vocalisation type was determined in the field using a Sound Level Meter (Digitech QM-1589) at a distance of *ca*.15 m from the bird. The original amplitude of vocalisation sound pressure level (SPL) at 1 m was then calculated using the formula of a 6 dB reduction for each doubling of distance between emission and reception^33^. The alarm calls of butcherbirds were also recorded by approaching active nests (N = 7) on foot. Butcherbirds’ nests were placed in average distance of 14.5 ± 1.5 m (mean ± SE, N = 7) from active noisy miners’ nests. Butcherbirds produce distinctive alarm calls in response to approaching humans (personal observation), and these vocalisations were recorded. Finally, eastern rosella chatter calls were recorded from calling individuals that were perched on trees (N = 5). Butcherbird and rosella calls were recorded using the same equipment and settings as those used for noisy miners (see above). Recorded calls were first grouped based on their type (chur, aerial, butcherbird or rosella) and the caller’s identity. For each individual/call type, we constructed a 2-minute playback sample using Raven Pro (v1.4; Cornell Laboratory of Ornithology). Each playback track consisted of 1 minute silence, followed by 20 seconds playback of the relevant stimulus, 20 seconds silence then finally another 20 seconds of playback. Only calls with a high quality of recordings were used.

### Playback experiment design

We assessed how noisy miner nestlings responded to broadcast of stimuli from 10 September to 30 November 2015. Fourteen different broods were exposed to playback, all when nestlings were 14 days post hatch. This age is just prior to fledging, so maximised the probability that nestlings were familiar with the different alarm calls and may have learnt to modify their behaviour accordingly. Prior to playbacks commencing, we installed a small tie-clip microphone under the nest cup (ECM-44B Sony, Japan) run by cables to a hide ~30 m away from the nest and connected to a solid-state audio recorder (Marantz PMD661, Japan), allowing recording of nestling vocalisations at high quality. Microphones were attached under the nest cup at least 1 hour before playbacks commenced to avoid any potential disturbance effects on nestling or attendant behaviour. No birds alarm called towards or interfered with the microphone over the experiment. We also placed a speaker (GG0191, JBL, USA) at a distance of ~5 m from the nest. It was connected to an audio player (Apple iPod) in the hide to allow playback of uncompressed wav files prepared above at a distance of ~30 m. Playback amplitudes were similar to the natural amplitude of the focal calls recorded for different calls (chur call: 83 dB, aerial alarm call: 89 dB) and remained consistent during all trials.

Playback experiments were conducted between 0700 to 1200 and 1400 to 1700 h. Each given ‘trial’ consisted of the playback of all 4 different call types, however presentation of call type order was randomised across nests. At the beginning of each trial, one or two observers entered the hide, although playbacks did not commence for a 10-minute period to allow broods to resume begging normally following any potential disturbance. After this period, playbacks were initiated when no adult birds were present within approximately 10 m of the nest and nestlings were vocalising. Begging data were recorded and measured only when broods were unattended for the duration of the trial. We commenced recording of all vocalisations at the nest before playback and continued until 5 minutes after playbacks had ceased in uncompressed WAV format (44.1 kHz, 16 bits) for later analysis. Four different stimuli were presented sequentially within each trial: (1) intraspecific chur calls, (2) intraspecific aerial alarm calls (3) butcherbird alarm calls, and (4) rosella chatter calls (a non-alarm vocalisation). Within a given trial, there was an interval of at least 30 minutes between playback of each call type to allow begging activity of broods to return to normal. The playbacks utilised multiple exemplars of the same call type that were recorded from different individuals to avoid pseudoreplication (chur call: N = 17, aerial alarm call: N = 10, butcherbirds call: N = 7 and rosella call: N = 5). Our aim was to broadcast at least one complete trial to each of the fourteen different broods used in the study. However, we aborted a given playback of a given stimuli type within a trial if an adult bird physically visited the nest with food (N = 10 playbacks) or if we detected an additional call being given by a free-living bird (e.g., a chur or aerial call was heard) during the playback period (N = 5 playbacks). Aborted playbacks were excluded from the analysis, and that trial repeated at a later time. However, we retained and included playbacks from incomplete trials that were not interrupted, being an additional 5, 10, 4 and 5 replicate playbacks for the chur alarm call, aerial alarm call, butcherbirds alarm call and rosella chatter call, respectively. Thus, while 14 broods were used in the study, total sample size for any given stimuli was higher through inclusion of repeat playbacks, leading to a final sample size of 19 chur call, 24 aerial alarm, 18 butcherbird alarms and 19 rosella chatter calls. As explained below, we accounted for repeat sampling by including nest identity as a random factor in statistical models.

### Acoustic analysis of brood begging in response to stimulus presentation

**Figure 2:**
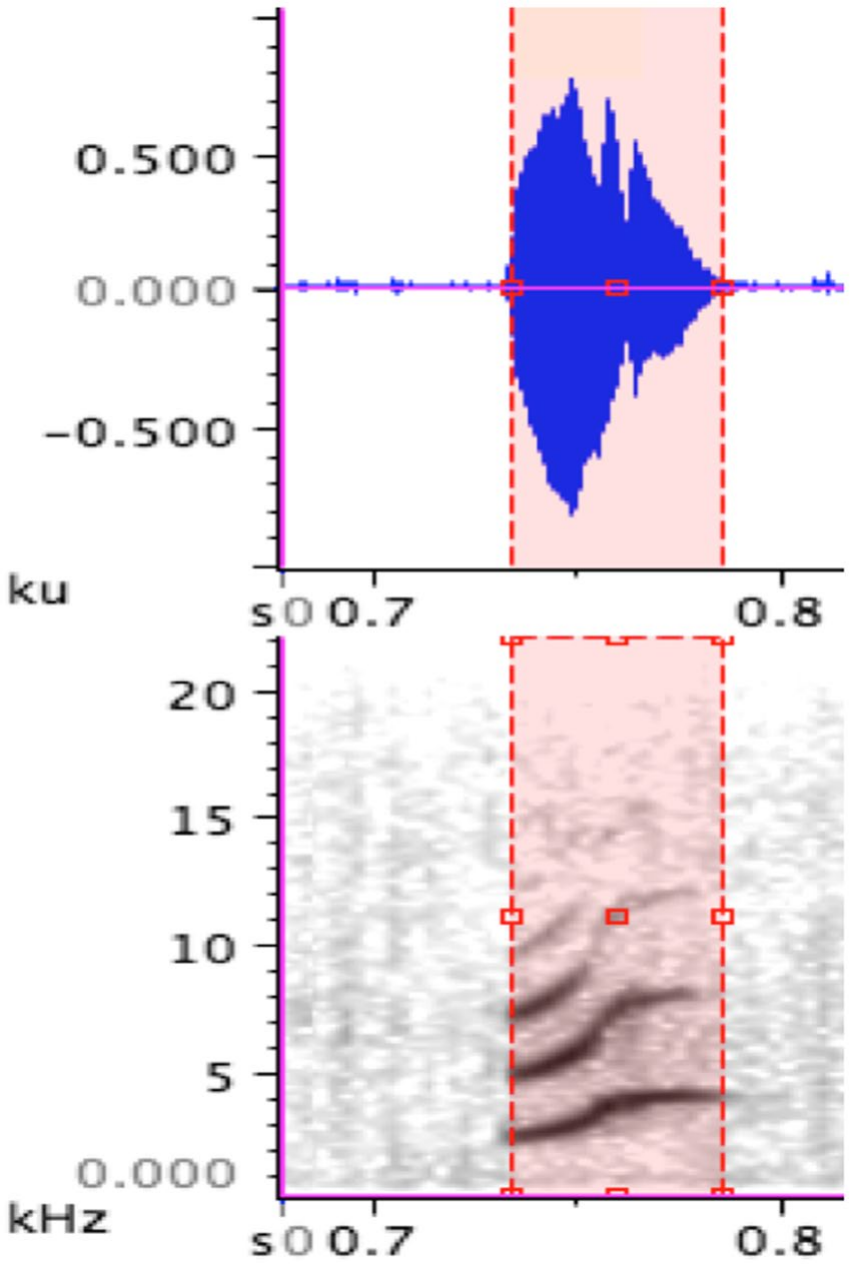
Sample spectrogram showing an example begging bout and how it was measured in Raven software.

Recorded begging calls were analysed in Raven Pro 1.4 (Cornell Laboratory of Ornithology). All spectrograms were constructed with a 256-point, 172 Hz grid spacing, Hanning window function with overlap set at 75% and a 3 dB Filter Bandwidth of 248 Hz. Calls were then filtered (highpass 500 Hz) to remove background noise at frequencies lower than the focal vocalisations. By examining spectrograms of recordings of each trial in Raven, we first identified the exact time playback started, then counted the number of begging bouts that occurred 10 seconds prior to playback commencing, and also during the first 10 seconds during playback (Fig. 1). After the 2 minutes playback was completed, we then identified suppression time as the interval between the end of playback and the start of nestling begging. For each playback, this time was identified on corresponding spectrogram in Raven software. We again counted the number of begging bouts per 10 seconds starting from the first begging call for this period. Each begging bout consisted of a single syllable that was given repeatedly as shown in Fig. 1a. We measured acoustic properties of 5 immediate bouts of begging before playback of stimulus (Fig. 1a) and 5 immediate begging bouts after playback of stimulus. However, in 24 playbacks (out of 80), acoustic properties of begging bouts could not be measured due to low quality of recording. Therefore the final samples size for begging bouts before and after playback were N = 376 (i.e. 376 begging bouts before and 376 after playbacks). If any of these five begging bouts were not clear enough for measurement, such as there were additional signals occurring at the same time, we measured the next available begging bout. The measurements were: (1) call amplitude (the root-mean-square amplitude of the selected part of the signal). (2) frequency at loudest amplitude (kHz) (or maximum frequency) and (3) length of each begging bout as the time differences between the starts and the end of a bout as shown in Fig. 2.

### Spectrographic cross-correlation to determine stimuli similarity

We used spectrographic cross-correlation (SPCC) to quantify the similarity of stimuli tracks, both within and between call types. We first used Sample Manager 3.2.0 (AudioPhile Engineering, USA) to convert stereo audio files to a mono audio file. Calls were then bandpass-filtered at 1000–22050 Hz^28^ and normalised before spectrograms were constructed with a 256-point, 172 Hz grid spacing, Hanning window function and overlap set at 75%, with 3 dB Filter Bandwidth of 248 Hz. We then compared spectrograms using the batch correlator tool in Raven Pro 1.4^34^. We obtained the peak correlation score for each pair of calls, which vary between 0, (for orthogonal signals) to 1 (identical signals)^34^.

### Statistical analyses

To test if the begging rate during test periods was similar to begging rate prior to playbacks commencing, we fitted a generalized linear mixed effects model (GLMM) with a poisson distribution including begging rate (e.g. number of begging bouts per 10 seconds) as the response variable and measurements time (control phase/during playback) as a fixed effect. Similar GLMMs were fitted to test whether stimulus type (chur, aerial, butcherbird or rosella) predicted the strength of response, the probability of begging being suppressed, the length of suppression time and the begging rate after suppression. In these models, the stimulus type (i.e. call type) was entered in the model as a fixed effect and begging rate, probability of suppression and the time of suppression as response variables. Response variables had either poisson (begging rate and suppression time) or binominal (probability of suppression) distributions. In all models playback order and nest identity were included as random effects. To assess if the effect of a fixed term (e.g. call type) was significant in a given model, we compared the model with an intercept-only model using likelihood ratio tests (LRTs). If LRTs were significant, we then performed *post hoc* Tukey tests to investigate within subject (e.g. within call types) differences.

We aimed to examine how acoustic properties change in response to the exposure to stimuli. To do this, we first averaged measured characteristics of 5 begging calls before and after playback for each playback trial. We then calculated the changes in these mean begging properties for each trial (e.g. after playback minus control). To reduce the dimensions of begging properties, we conducted a principal component analysis (PCA). To test if the begging structure varies in relation to stimulus type, we fitted a generalized linear model (GLM) with a gaussian distribution with call type as a fixed effect and the first extracted component of the PCA as the response variable. To examine the SPCC similarities between and within call types, we fitted additional GLMMs. In the models the response variables were pairwise similarity between different calls, including the terms of interest (e.g. call types) as fixed effects and playback order and nest identity as random effects. To test the significance of the term of interest, GLMMs were compared with a reduced model that contained only the intercept term and random effects using likelihood ratio tests (LRTs). To examine within subject differences a *post hoc* test Tukey test was used. All statistical analysis tests were performed in the R statistical language and environment^35^ using the *lme4* package^36^.

## Results

### Changes in begging rate in response to different playback stimuli

**Figure 3:**
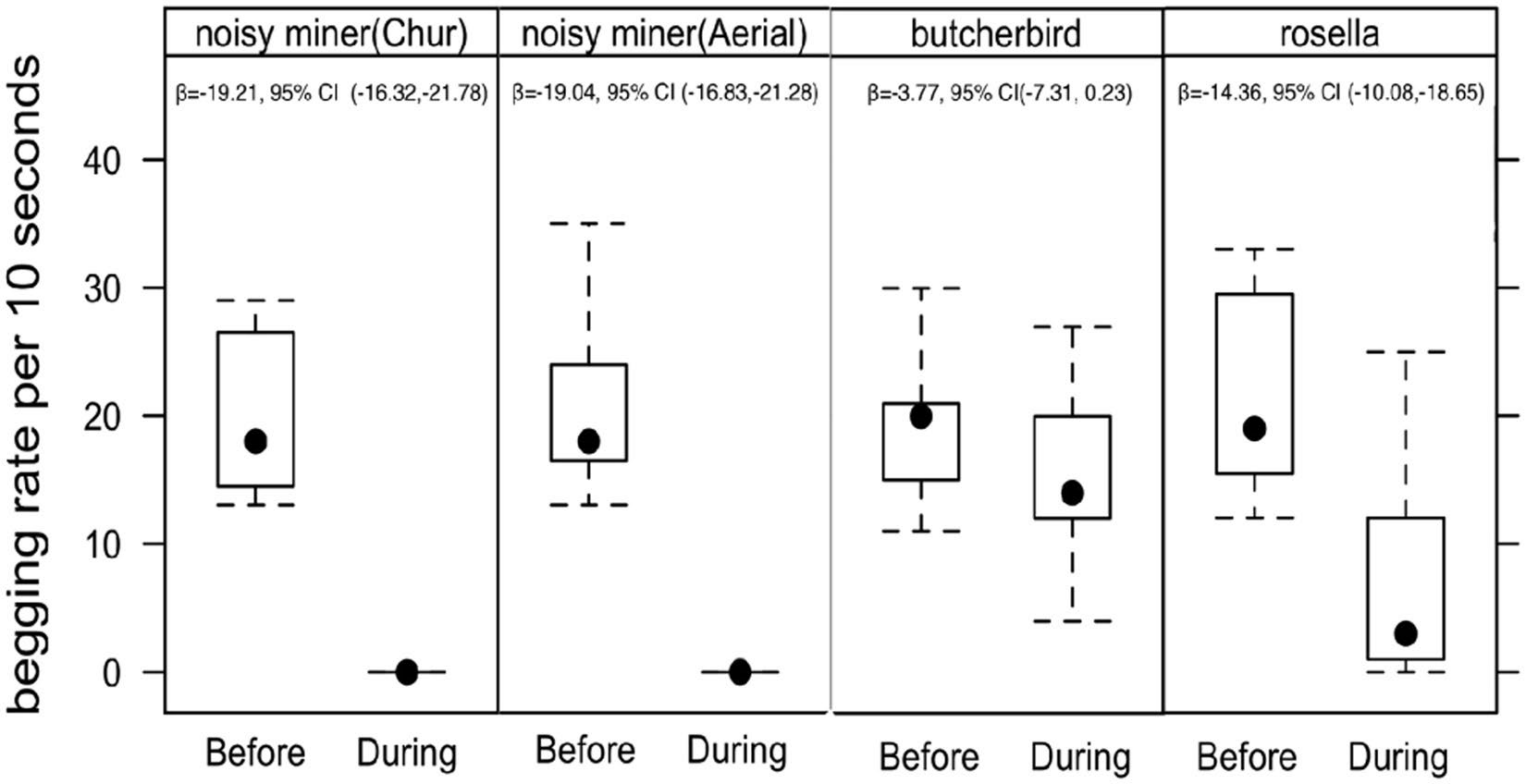
Begging rates of nestling noisy miners before and during palyback of different calls. For each call type, effect size and 95% CIs are given in the corresponding panel. In both before and during playback, begging rate is the number of begging bouts in a 10-second time frame.

The rate of begging call production (call syllables per 10 seconds) in control periods prior to stimulus playback did not differ between broods exposed to different playback types (GLMM: χ^2^_3_ = 2.28, p = 0.51). There was a significant decrease in begging rates in response to aerial alarm calls (GLMM, χ^2^_1_ = 87.14, p < 0.001), chur alarm calls (GLMM, χ^2^_1_ = 66.88, p < 0.001) and rosella chatter calls (GLMM, χ^2^_1_ = 24.86, p < 0.001) (Fig. 3). In contrast, butcherbirds calls did not provoke a significant decline in the begging rate of nestlings (GLMM, χ^2^_1_ = 10.82, p = 0.07, Fig. 3).

When comparing the comparative strength of changes in begging rate during the playback period across different call types, there was a significant difference (GLMM, χ^2^_3_ = 4.825, p < 0.001). When call types were compared using a *post hoc* test, a greater reduction in the begging rate in response to aerial alarm call (*post hoc* GLMM, β = −12.8 ± 1.96, p = 0.0001), chur alarm call (*post hoc* GLMM, β = −14.35 ± 2.07, p = 0.0001) and rosella chatter call stimuli (*post hoc* GLMM, β = −0.47 ± 2.06, p = 0.0001) was apparent relative to butcherbird calls (Fig. 3). Similarly, call type predicted the probability of suppression of begging (GLMM, χ^2^_3 = _70.21, p < 0001). The probability that nestlings cease begging was significantly lower in response to butcherbird calls compared to both aerial alarm (*post hoc* GLMM, β = 0.87 ± 0.10, p < 0.001) and chur alarm calls (β = 0.84 ± 0.10, p < 0.001).

**Figure 4:**
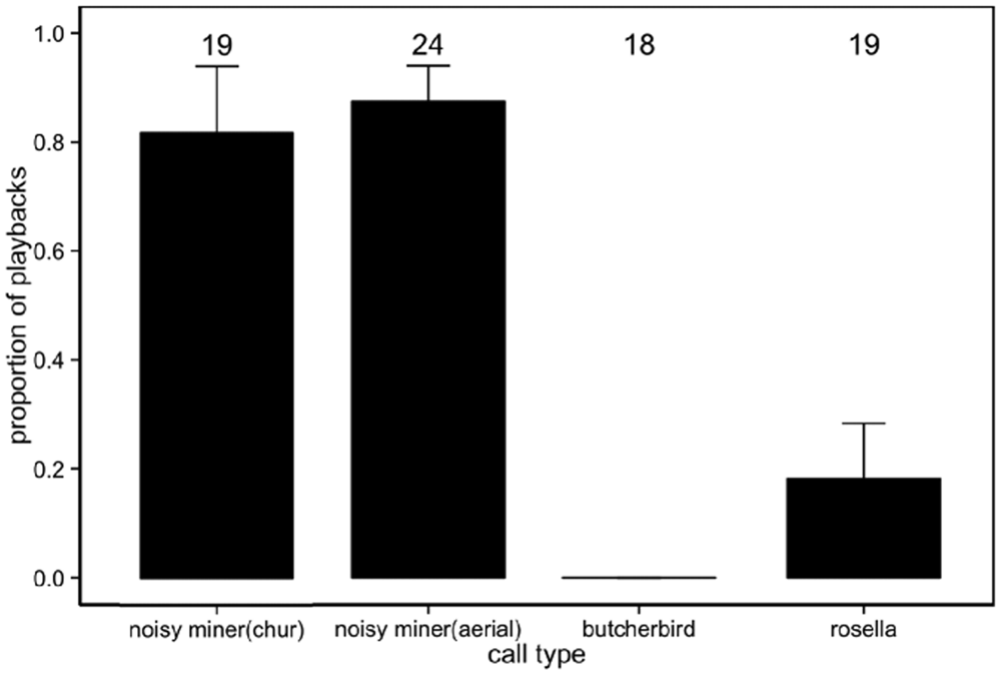
Mean ± se of proportion of playbacks that nestling noisy miners suppressed vocalisation in response to different playback stimuli. Numbers above bars represent sample sizes for each playback type.

While there was significantly higher probability that nestlings supressed begging vocalisations in response to intraspecific alarms calls than to interspecific calls (GLMM, χ^2^_1_ = 66.26, p < 0.001; Figs 1–4), the proportion of times that nestlings suppress begging was not different for two types of intraspecific alarms calls (*post hoc* GLMM, β = 0.03 ± 0.10, p = 0.9; Fig. 4) and two interspecific calls (*post hoc* GLMM, β = −0.21 ± 0.10, p = 0.20; Fig. 4).

**Figure 5:**
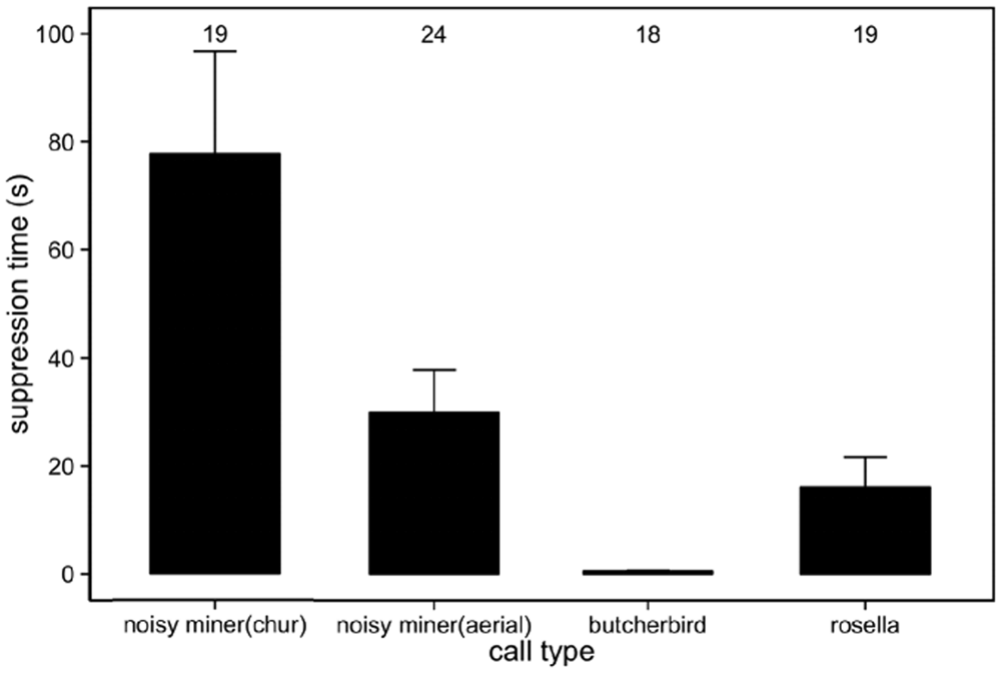
Mean ± se suppression time of nestling noisy miners to different playback stimuli. Numbers above bars represent sample sizes for each playback type.

Further, suppression time was different in relation to call type (GLMM, χ^2^_3_ = 2112.5, p < 0.0001). All pairwise comparisons of suppression time differed significantly (Fig. 5; Table 1). Nestlings suppressed begging vocalisations for the longest time in response to chur alarm calls, followed by aerial alarms and then rosella chatter calls. Suppression time in response to butcherbirds was the shortest among all call types (Fig. 5, Table 1). When nestlings resumed begging again, begging rate remained independent of the stimulus type that they were exposed to during playbacks (GLMM, χ^2^_3_ = 1.49, p = 0.68).

**Table 1:** Pair-wise comparisons of suppuration time of begging by nestling noisy miners in response to playback stimuli (call type A-call type B).

Call Type A	Call Type B	β	SE	z	*p*
butcherbird	aerial	−4.15	0.33	−12.41	<0.001
chur	aerial	0.91	0.04	19.52	<0.001
rosella	aerial	−0.69	0.06	−10.09	<0.001
chur	butcherbird	5.07	0.33	15.18	<0.001
rosella	butcherbird	3.46	0.33	10.25	<0.001
rosella	chur	−1.60	0.06	−25.36	<0.001

### Changes in the spectral structure of begging vocalisations during playbacks

**Figure 6:**
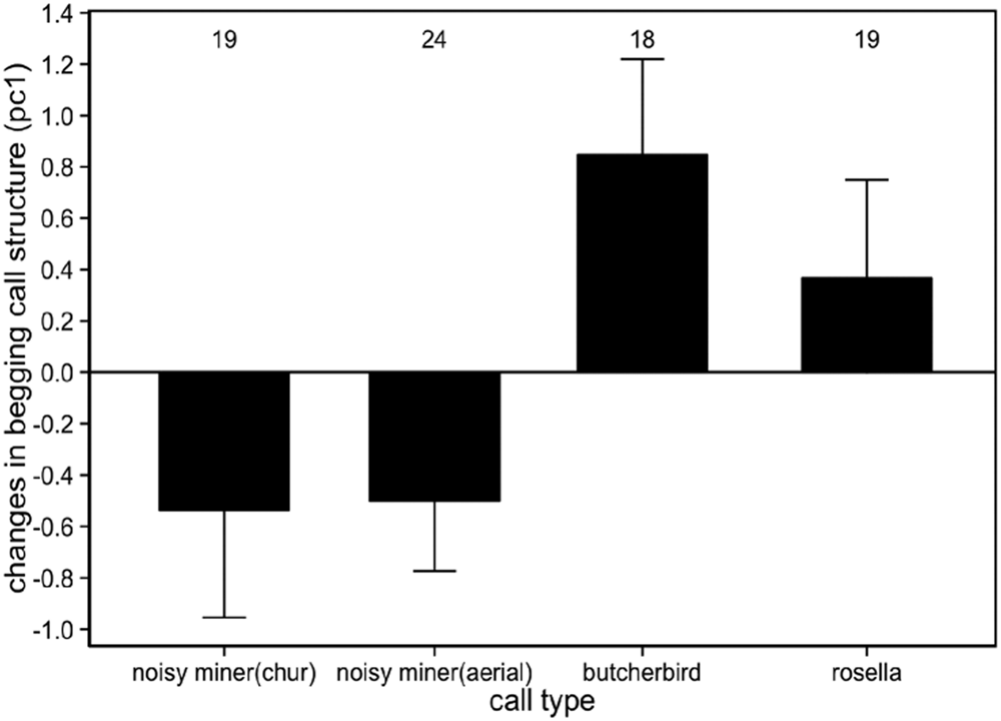
Mean ± SE of variations in the acoustic structure (represented by PC1) of nestlings begging after playback of different stimuli. Numbers above bars represent sample sizes for each playback type.

We first calculated the degree of change (the values 10 seconds after playback minus the values 10 seconds before playback) of three begging characteristics (e.g. amplitude, frequency at loudest amplitude and duration) and then extracted principal components with eigenvalues larger than 1 through PCA. Only the first component (hereafter: PC1) had an eigenvalue that was greater than 1. PC1 was positively correlated with changes in begging call amplitude (Pearson correlation = 0.69, df = 78, p < 0.0001), frequency at loudest amplitude (Pearson correlation = 0.73, df = 78, p < 0.001) and begging duration (Pearson correlation = 0.87, df = 78, p < 0.001). Therefore, larger PC1 values reflect increasing call amplitude, frequency at loudest amplitude and duration during post-stimulus period relative to control phase (before playback). PC1 was significantly different in relation to stimulus types (GLMM, χ^2^_3_ = 15.5, p < 0.001). Nestlings begged with lower amplitude and frequency, and shorter duration during post-stimulus period relative to control phase (before playback) for both aerial and chur calls (*post hoc* GLMM, aerial: β = 1.06 ± 0.32, p < 0.001; chur: β = 1.09 ± 0.33, p < 0.001; Fig. 6), however, no differences was apparent between these two types of intraspecific calls (*post hoc* GLMM, β = 0.02 ± 0.31, p = 0.99). Further, variations of begging structure did not significantly differ in response to two interspecific calls (*post hoc* GLMM, β = 0.37 ± 0.33, p = 0.68).

### Similarity of stimulus signals across different vocalisation types

We compared the similarity both between and within the different call types used as stimulus during these playbacks using spectrographic cross correlation (SPCC). As expected, calls from different birds of the same call type (e.g. within-stimulus) were significantly more similar than between-stimulus (GLM, χ^2^_1_ = 6.46, p < 0.001). The similarity of calls varied in relation to call type (GLMM, χ^2^_5_ = 0.29, p < 0.001). When between-stimulus calls were compared, both chur and aerial alarm calls were significantly more similar to rosella chatter calls than to butcherbird calls (*post hoc*, GLMM, aerial: β = 014 ± 0.002, p < 0.001; chur: β = 0.09 ± 0.002, p < 0.01). However, two different intraspecific calls were not significantly different in their similarity to rosella chatter calls (*post hoc*, GLMM, β = −0.001 ± 0.003, p = 0.99) or butcherbird calls (*post hoc*, GLMM, β = 0.003 ± 0.001, p = 0.23).

## Discussion

This study provides experimental evidence that nestling noisy miners attended to alarm signals given by conspecifics and suppressed their begging on hearing those signals alone, broadening the taxa in which this result has been documented^7,8^. This study was further able to demonstrate that noisy miner nestlings responded more strongly to intraspecific rather than interspecific alarm calls, with the strength of response in terms of suppression time varying significantly for the two different intraspecific alarm calls broadcast. This suggests that nestlings can differentiate between these functionally referential intraspecific alarm calls. Further, among the two interspecific stimuli played back to nestlings, nestlings responded only to the call that was more similar to intraspecific, despite this not being a vocalisation specific to the presence of predators and thus being associated with danger. Our analysis of call similarity based on spectrographic cross-correlation suggested that this response is most likely due to recognition error by broods, given that rosella chatter calls are somewhat similar to noisy miner alarm calls.

Nestling noisy miners showed a strong response to intraspecific alarm calls, and in over 80% of playbacks, broods suppressed vocalisation upon hearing these alarm calls regardless of their type. These results suggest that nestling noisy miners could effectively respond to intraspecific alarm calls nearby and reduce their predation risk, adding to very rare examples of this behaviour in other avian taxa (e.g. white-browed scrubwren, *Sericornis frontalis* ^8^).

Although nestlings showed similar, suppressive responses to chur and aerial alarm calls, nestlings ceased begging for a longer period in response to chur calls rather than aerial alarm calls of adult noisy miners. In this system, chur alarm calls warn of ground predators, often in response to approaching potential threats or when a disturbance is detected within the species’ colony^20^. This call attracts colony members to the area and elicits mobbing behaviour^28,30^. Previous studies have found that this call is given in response to foxes (*Vulpes vulpes*), feral cats (*Felis catus*) and perched predatory birds in the study area^20,28^. Therefore, chur calls usually signal a more urgent threat to nestlings, such as a raptor that has perched nearby, than aerial alarm calls do where the flying predator is quickly gone from the immediate area^20,28–30^.

Given that the response of nestlings in terms of the probability of suppression and the reduction in begging rate did not differ when exposed to two intraspecific alarm calls, nestling response is likely an immediate and innate reaction to these calls. However, the functions of chur and aerial alarm calls suggest that aerial alarm calls encode a less urgent threat compared to chur alarm calls, thus it is likely that nestlings have learnt to associate chur calls with immediate and greater danger and fine-tune their response based on the information that they have gained during nestling period.

In many species, aerial predators are usually more reliant on visual cues rather than acoustic signals^37^. Given this, in species with concealed nest sites such as the noisy miner, aerial predators are less threat than ground predators. This might explain why the suppression time is longer in response to chur calls. Differentiating between alarm calls and showing adaptive adjustments in responses to these has rarely been reported in nestling birds. To date, only white-browed scrubwren nestlings have been shown to respond to ground alarm calls more intensely than to aerial alarm calls, with ground predators posing a greater threat to the nestlings^16^.

Nestlings modified their begging acoustic structure following exposure to either aerial or chur alarm call stimuli from conspecifics. They begged at a lower amplitude, lower frequency and for a shorter time after they resumed begging following exposure to these alarm calls. Generally, nestling vocalisations can attract predators to the nest area^3,38^ and there is evidence that the acoustic properties of begging are associated with the degree of predation risk. For example, species with a lower amplitude of begging call experience lower predation risk^38^ because lower amplitude reduces the locatability of broods by predators. Therefore, it may be possible that altered acoustic properties provides a reduction in the risk of broods being detected by predators, acting as a passive defence that makes begging calls more difficult for predators to locate^37^. However, if the begging calls are honest signals of need, these changes can also influence the amount of food that nestlings would receive from nest attendants^39–41^, so such changes would likely be transient and short-term in nature. This implies that acoustic adjustment of begging calls to intraspecific alarm calls represents a trade-off between avoiding danger and receiving sufficient food, and the intensity of this response perhaps depends on the nestlings’ hunger level.

Nestling noisy miners responded differently to the two different conspecific calls used in this experiment. They largely ignored the alarm calls of the sympatric grey butcherbird. Similar to noisy miners, butcherbirds bids also mob potential nest predators, even in mixed mobbing group with noisy miners^26,31^. Further, butcherbirds are closely associated with noisy miners in the studied colonies and usually built their nest at a close distance to those of noisy miners (see methods). Therefore, nestling noisy miners have the opportunity to learn the link between Butcherbird alarm calls and a potential threat during their developmental period before fledging, however the results show that this did not occur. Despite this, it cannot be ruled out that nestlings will learn to respond to butcherbird alarms when they are older. Thus, for understanding the response of noisy miners to interspecific alarm calls, it would be useful to examine the response of adults and fledglings to butcherbird alarm calls.

In contrast to their response to butcherbirds, nestlings showed a partial response to sympatric rosella chatter calls, which are not used as alarm calls but rather function to attract flying groups to perch near the caller^26^. The cost of inappropriate responses to non-alarm calls is probably minor compared with the cost of not responding to honest and accurate alarm call signals. There is evidence that various sensory pathways enable individuals to acoustically discriminate between familiar and unfamiliar individuals^42^ and focus their attention on sound features of their own species’ song^43^, including adult noisy miners^44^. These abilities can then be used to improve the learning of their own species’ signals from the many interspecific calls and signals in the surrounding acoustic environment^7^. Contrary to nestling responses, adult noisy miners usually ignore rosella calls (Barati, unpublished data), therefore it is likely that miners do eventually learn to ignore these irrelevant stimuli as they mature. This has been confirmed in some species. For example, offspring of the white-browed scrubwren adaptively change their response to alarm calls as they grow and show stronger responses to aerial alarm calls after they have fledged^10^.

Interestingly, the changes in begging acoustic properties occurred only in response to intraspecific alarm calls, not interspecific signals. Broods did change their begging rate following exposure to rosella chatter calls, but they did not modify the acoustic structure of their begging calls, suggesting that the response to rosella calls is probably an immediate short-term response, most likely due to recognition confusion and a failure to completely discriminate interspecific calls from intraspecific alarm calls. Nestlings may learn rapidly to discriminate intraspecific alarm calls from similar calls of other species, but this seems unlikely given the broods were close to fledging at the time of this experiment. How noisy miners distinguish between different alarm calls and the role of learning in this process remains unclear, and further research is required using nestlings that have been isolated from exposure to some calls to tease these factors apart.

In conclusion, we show that nestling noisy miner broods suppressed begging vocalisations in response to intraspecific alarm calls in a manner consistent with adaptively reducing their predation risk. Our results confirm previous findings that nestlings can lower the risk of predation and other threats using likely innate responses^45,46^. Moreover, we demonstrate herein that nestlings can, either innately or through learnt responses, discriminate between two different conspecific alarm calls that signal different relative threats. The study thus provides experimental evidence that the response of offspring to a particular intraspecific alarm call in this system is linked to the degree of danger that particular signal is likely to indicate. We also provide as far as we are aware the first empirical evidence that nestlings adjust the structure of their begging calls when they are exposed to changes in predation risk, again likely a response that reduces the risk of detection by a predator. Given that sociality tends to be associated with complex repertoires in social species including the noisy miner^20^, research investigating interspecific alarm call recognition in less social species would provide an enlightening comparison.
